# COVID-19 as Cause versus Trigger for the Collapse of Capitalism

**DOI:** 10.1177/0020731420977711

**Published:** 2020-12-09

**Authors:** Howard Waitzkin

**Affiliations:** 1 Department of Sociology and Health Sciences Center, 1104 University of New Mexico, Albuquerque, New Mexico, USA

**Keywords:** COVID-19, global economy, capitalism, pandemic, ideology, economics

## Abstract

According to the official narrative of COVID-19, the pandemic has caused the global capitalist economy to collapse, or at least to enter a deep recession and possibly a great depression. Assigning blame to a virus takes attention away from the structural contradictions and instabilities of capitalism that would have led to a crash in any case. This narrative also helps justify non-evidence-based public health policies, including lockdowns, travel bans, closed schools and factories, and forced quarantines of large populations rather than individuals and clustered groups who harbor the infection. Advantages of such drastic measures happen primarily in countries that did not prepare adequately, that did not respond quickly enough with more focused measures to test and isolate people infected with the virus, and that have health care systems either organized by capitalist principles or suffering cutbacks and privatization as a result of capitalist economic ideologies, such as austerity. Authoritarian tactics purportedly intended to protect public health pave the way to antidemocratic rule, militarism, and fascism. These harsh policies also exert their most adverse effects on poor, minority, incarcerated, immigrant, and otherwise marginalized populations, who already suffer from the worsening economic inequality that global, financialized capitalism has fostered.

## Narrative of Economic Collapse During COVID-19

The official narrative of COVID-19 states that the pandemic has caused the global capitalist economy to collapse, or at least to enter a deep recession and possibly a great depression, but is that correct?

A more accurate interpretation is that the pandemic has triggered a collapse that was going to happen anyway. For many years, the global capitalist economy has been crisis-ridden, unstable, and “bubbly…subject to blowups.”^1^ In August 2019, the interest yield on a 10-year US Treasury bond fell below that of a two-year bond. This inversion, indicating a marked decline in investors’ confidence in long-term earnings, has preceded every recession since the 1950s. These and other economic trends led the editors of *Monthly Review* to predict: “There is now little doubt that the world economy is on the verge of a recession after a long sluggish recovery from the Great Financial Crisis of 2007–09…. In this instance, however, there lurks a bigger fear, the possibility of a financial Armageddon on the level of the Great Financial Crisis of 2008—or worse.”^2^

Conveniently, the COVID-19 narrative assigns blame for the economic crash to a virus, taking attention away from the structural contradictions and instabilities that would have led to a crash in any case, as predicted for many months before the pandemic began. The global capitalist economy has switched to the expansion of finance capital and away from production of useful goods and services. Financialization now creates “fictitious capital” such as packages of risk, derivatives, and futures. These fictional financial instruments involve gambles on the future valuation of an imaginary reality that does not correspond to any concrete economic good, service, or property. Global markets in financial instruments therefore become a more elite version of gambling that traditionally takes place in poker games, casinos, and racetracks.

Creation of fictitious capital and accumulation of capital through gambling create a vulnerability to burst financial bubbles and crashes like that of 2008. That particular crash derived from the collapse of collateralized loan obligations: financial instruments that bundled housing loans for investment in global financial markets. As the COVID-19 pandemic worsened, large investors spurred the rapid decline in prices of stocks and fictional financial instruments, as they rapidly sold off holdings that had become overvalued. Later, global stock markets have become more volatile while economic recession has deepened, throwing millions of people into unemployment, housing insecurity, and hunger. Blaming a virus for the crash mystifies the economic contradictions actually responsible for the abrupt end of the latest capitalist bubble.^3^

## Non-Evidence-Based Public Health Policies Versus Epidemiology 101

The false narrative of viral infection as the cause of economic collapse also justifies public health policies that have little or no scientific basis. Such influential but mostly non-evidence-based public health policies include lockdowns, travel bans, closed schools and factories, and forced quarantines of large populations rather than individuals and clustered groups who harbor the infection.^4^ For instance, multiple research studies of school closures during this pandemic and prior epidemics show that risks for mortality due to loss of health workers who cannot access alternative childcare balance or outweigh the benefits of reduced contagion through children.^5^

The advantages that arise from these drastic measures happen primarily in countries that did not prepare adequately for the pandemic, that did not respond quickly enough with more focused measures to test and isolate people infected with the virus, and that have health care systems either organized by capitalist principles or suffering cutbacks and privatization in recent years because of capitalist economic ideologies, such as austerity. Unprepared countries, especially the United States, have not even tried to implement the procedures that we learn in Epidemiology 101:

- Quickly identify individuals and groups at high risk, especially those who have traveled from specific geographical areas where an epidemic already has spread.
- Test those individuals and groups to find out if they have the infection.
- Strictly quarantine individuals and groups who are waiting for test results and/or who test positive.
- Make sure those in quarantine have enough food, toilet paper, money, and other necessities of life so that they do not try to escape from the quarantine.
- Make sure those who test positive receive free or extremely inexpensive medical treatment and surveillance, with a clearly established end point to determine when they are ready to leave quarantine.
- Convey honest and transparent information to the general population about specific geographical locations with prevalent infection, so people can get tested if they have been there and can avoid the areas if they have not been there.
- Provide financial support for anyone who suffers economic hardship during an epidemic, especially if a person becomes unemployed, but also if there are other sources of vulnerability such as poverty, disability, ethnic/racial minority status, young or old age, recent migration, or chronic illnesses.
- Initiate economic policies that preserve people’s jobs by paying employers to continue wages and benefits while people cannot work, rather than allowing large numbers of people to be laid off and face the insecurity of uncertain future employment.^6^

Health professionals and politicians who advocate for more general, population-based interventions, such as lockdowns, either didn’t take Epidemiology 101 and therefore do not have a clue how to respond to epidemics, or they did take Epidemiology 101 and understand that their political–economic system does not provide a public health infrastructure that permits the above procedures, either early enough during an epidemic or at all—for instance, in the United States, which has lacked a viable public health infrastructure for decades, at least since the drastic cutbacks achieved as part of neoliberal policies starting in the 1980s.

## Harsh Consequences of Harsh Public Health Policies

Countries that did prepare, did test and isolate infected people, and have health care systems organized around universal access to services and a well-financed and organized approach to public health have done much better in controlling the pandemic, at least so far. Complete lockdowns generally have not occurred in these countries. Such harsh measures, with their devastating economic effects leading to massive unemployment, lost income and retirement accounts, and worsening food and housing insecurity, may seem reasonable for countries whose leaders did not act decisively at an early stage. But we should not delude ourselves about the scientific evidence for these measures’ effectiveness, nor about the viral pandemic itself as the cause of the overall economic collapse.

Although the health benefits of such policies remain dubious, the benefits for protecting what remains of global capitalism are substantial. As in 2008, a massive injection of tax-generated, public-sector funding to private corporations creates a socialism for the rich. This process includes a version of disaster capitalism, in which corporations receive public subsidies to protect the capitalist economy.^7^ Authoritarian tactics applied by some governments, purportedly to contain the pandemic and to protect the economy, pave the way to antidemocratic rule, militarism, and fascism. This path becomes especially dangerous when police and military forces get involved in repressive tactics justified by public health and humanitarian rationales. These harsh policies, purportedly implemented to protect the public’s health, also exert their most adverse effects on poor, minority, incarcerated, immigrant, and otherwise marginalized populations, who already suffer from the worsening economic inequality that global, financialized capitalism has fostered.^8^

## References

[1] Medlen CA. Free Cash, Capital Accumulation and Inequality. New York: Routlege; 2018:14.

[2] Notes from the Editors. *Monthly Review*. October 2019:71, no. 5. https://monthlyreview.org/2019/10/01/mr-071-05-2019-09_0/

[3] Toussaint E. The capitalist pandemic, coronavirus and the economic crisis. *Committee for the Abolition of Illegitimate Debt*. March 19, 2020. http://www.cadtm.org/The-Capitalist-Pandemic-Coronavirus-and-the-Economic-Crisis; No, the coronavirus is not responsible for the fall of stock prices. *Committee for the Abolition of Illegitimate Debt*. March 5, 2020. http://www.cadtm.org/No-the-coronavirus-is-not-responsible-for-the-fall-of-stock-prices; Waitzkin H. Revolution now: teachings from the Global South for revolutionaries in the Global North. *Monthly Review*. June 2017;10(2):18–36. https://monthlyreview.org/2017/11/01/revolution-now/

[4] Kavanagh M. Transparency and testing work better than coercion in coronavirus battle. *Foreign Policy*. March 16, 2020. https://foreignpolicy.com/2020/03/16/coronavirus-what-works-transparency-testing-coercion/; Ioannidis JPA. A fiasco in the making? As the coronavirus pandemic takes hold, we are making decisions without reliable data. *StatReports*. March 17, 2020. https://www.statnews.com/2020/03/17/a-fiasco-in-the-making-as-the-coronavirus-pandemic-takes-hold-we-are-making-decisions-without-reliable-data/

[5] Viner RM Russell SJ Croker H, et al. School closure and management practices during coronavirus outbreaks including COVID-19: a rapid systematic review. *Lancet Child and Adolescent Health*. April 6, 2020. https://www.thelancet.com/pdfs/journals/lanchi/PIIS2352-4642(20)30095-X.pdf; Cauchemez S, Ferguson NM, Wachtel C, et al. Closure of schools during an influenza pandemic. *Lancet Infect Dis*. 2009;9:473–481. https://www.ncbi.nlm.nih.gov/pmc/articles/PMC7106429/10.1016/S2352-4642(20)30095-X PMC7270629 32272089

[6] Saez E Zucman G. Jobs aren’t being destroyed this fast elsewhere. Why is that? *New York Times*. March 30, 2020. https://www.nytimes.com/2020/03/30/opinion/coronavirus-economy-saez-zucman.html

[7] Solis M Klein N. Coronavirus is the perfect disaster for ‘disaster capitalism. *Vice*. March 16, 2020. https://www.vice.com/en_in/article/5dmqyk/naomi-klein-interview-on-coronavirus-and-disaster-capitalism-shock-doctrine

[8] Kapczynski A Gonsalves G. Alone against the virus: class and inequality. *Boston Review*. March 13, 2020. http://bostonreview.net/class-inequality-science-nature/amy-kapczynski-gregg-gonsalves-alone-against-virus; Prins N. The global economy catches the coronavirus. *TomDispatch.com*. March 12, 2020. http://www.tomdispatch.com/post/176674/tomgram%3A_nomi_prins%2C_the_global_economy_catches_the_coronavirus/#more. For more on the impending global crisis of food insecurity caused by lockdowns as a claimed public health policy, see: Vijay Prasad. Hunger gnaws at the edges of the world. *Tricontinental: Institute for Social Research*. May 14, 2020. https://mailchi.mp/thetricontinental.org/hunger-gnaws-at-the-edges-of-the-world-the-twentieth-newsletter-2020?e=7aac4cc929; Pollan M. The sickness in our food supply. *New York Review of Books*. June 11, 2020. https://www.nybooks.com/articles/2020/06/11/covid-19-sickness-food-supply/

